# Atlanto-axial rotatory fixation in a girl with Spondylocarpotarsal synostosis syndrome

**DOI:** 10.1186/1748-7161-1-15

**Published:** 2006-10-16

**Authors:** Ali Al Kaissi, Farid Ben Chehida, Hassan Gharbi, Maher Ben Ghachem, Franz Grill, Klaus Klaushofer

**Affiliations:** 1 Ludwig-Boltzmann Institute of Osteology at the Hanusch Hospital of WGKK and AUVA Trauma Centre Meidling, 4th Medical Department, Hanusch Hospital. Heinrich Collins Str. 30 A-1140, Vienna, Austria; 2 Department of Paediatric Orthopaedic Surgery-Children Hospital of Tunis, Jabari, 1007 Tunisia; 3 Center of Radiology-Department of Imaging Studies-Ibn Zohr Institute, Tunis, City El-Khadra 1003, Tunisia; 4 Orthopaedic Hospital of Speising, Paediatric Department, 109-Speisninger Str. Vienna-1130, Austria

## Abstract

We report a 15-year-old girl who presented with spinal malsegmentation, associated with other skeletal anomalies. The spinal malsegmentation was subsequently discovered to be part of the spondylocarpotarsal synostosis syndrome. In addition, a distinctive craniocervical malformation was identified, which included atlanto-axial rotatory fixation. The clinical and the radiographic findings are described, and we emphasise the importance of computerised tomography to characterize the craniocervical malformation complex. To the best of our knowledge, this is the first clinical report of a child with spondylocarpotarsal synostosis associated with atlanto-axial rotatory fixation.

## Background

There have been more than 20 clinical reports of the Spondylocarpotarsal synostosis syndrome, (SSS), a condition in which patients primarily present with scoliosis/kyphoscoliosis. It is characterised, by failure of normal spinal segmentation, resulting in block vertebrae and fusion of posterior elements. Carpal and/or tarsal coalition, pes planus, dental enamel hypoplasia, decreased range of motion or dislocation of the elbow, renal anomalies, and hearing loss, are additional features. Our patient presented with scoliosis, and later, with persistent torticollis. Radiographic evaluation of the cervicocranium, which is traditionally based on the anteroposterior (open-mouth) and lateral spine radiography, was not contributory. CT scans revealed atlanto-axial rotatory fixation.

Atlanto-axial rotary fixation, (AARF) has been reported in connection with Marfan syndrome. Radiographic analysis of patients with Marfan syndrome has shown that, atlantoaxial rotatory subluxation can also occur. An increased atlanto-axial translation, larger odontoid height, and basilar impression are more prevalent in the Marfan-population compared to age-matched controls [1]. Some clinical reports describe the association of Spondylocarpotarsal synostosis syndrome and cervical malformations, [2,3]. The cause of SSS is unknown, although autosomal recessive inheritance has been suggested. We herein reported a patient with SSS, with the additional atlanto-axial rotatory fixation. To the best of our knowledge neither AARF nor the role of computerized tomography to investigate the craniocervical junction, have been reported in patients with SSS.

## Case presentation

A) The proband presented with thoracic scoliosis (Cobb angle of 85 degree) and torticollis. She was the product of an uneventful gestation, with a birth weight of 2900 g, a length of 48 cm, and a head circumference occipital-frontal circumference (OFC) of 31 cm. Her mother was a 29-year-old gravida 3, abortus 0 (term to signify the maternal obstetrical history, i.e. history of three pregnancies with no history of spontaneous abortion, it has specific significance in genetically determined disorders), married to a 33-year-old first-degree relative. She had no history of serious illness, and her developmental history was almost within the normal limits, apart from a delay in walking, which commenced at the age of 2 years.

**Figure 1:**
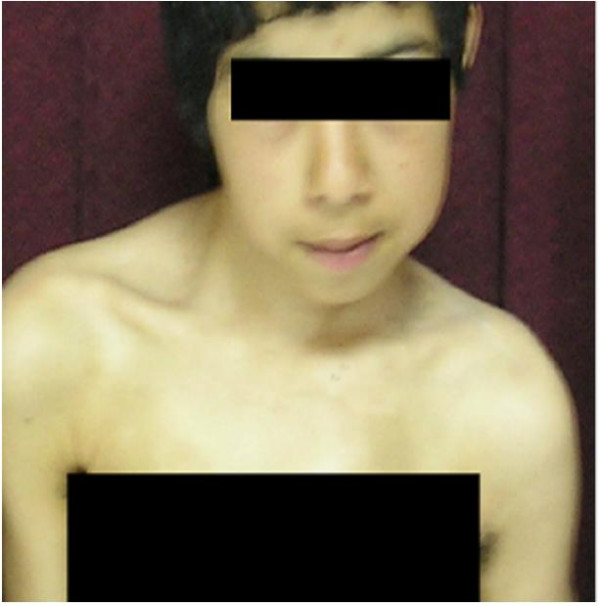
Phenotype.

Examination at the age of 15 years revealed (fig. 1), a height of 138 cm (-3SD), a weight of 49 kg (-1SD) and a OFC of 52 cm, which was normal. The craniofacial features were, prominent eyes, hypertelorism, facial asymmetry, retrognathia, coarse and thick scalp hair, ptyrigium colli, (more marked on the right side). Ligamentous laxity, long thin limbs, short trunk, pes planus and significant cervicothoracic scoliosis were the most prominent Orthopaedic abnormalities encountered. Neurological examination showed no deficits. Hearing, vision and intelligence were normal. Her torticollis was not accompanied by pain, and it was to the left side. On attempting to turn her head to the right, she was unable to pass the midline. There was no associated spasm of the sternomastoid muscle on the opposite side of the torticollis, and there was no shortening of the sternomastoid muscles.

The child underwent a series of investigations, which included, a urine screen for metabolic disorders, a complete blood count, chromosomal analysis, basic hematological tests, plasma and urinary aminoacids assays. The results of these tests were normal. The type of scoliosis in our patient is malignantly progressive early in life and steadily during growth (poor prognosis). It was therefore treated through stabilisation of the spine. This was done by a posterior in situ fusion. The fused area incorporated the entire bar and extended one mobile level above and below the bar. Clinical and radiological follow up was mandatory to further assessing the condition. On the other hand, the suggested plan for the treatment of AARF is either a transoral decompression followed by a posterior fixation or the same procedure without posterior fixation

B) The radiographic examination:

Renal and pelvic ultrasounds showed normal genito-urinary system, and her ovaries, tubes, uterus were normal.

* The Wachenheim clivus line (a method to evaluate and assess craniocervical junction abnormality/injury), a line drawn along the posterior aspect of the clivus toward the odontoid process. An abnormality is suspected when this line does not intersect and or/not tangential to the odontoid process. [4].

** Chamberlain's line joins the hard palate to the posterior lip of the foramen magnum. Basilar impression is defined as being present when the tip of the dens projects more than 5 mm above Chamberlain's line [5].

## C) Discussion

Atlanto-axial subluxation is a rotational disorder of the atlanto-axial joint, that results in either limited rotation of the neck, or, in rare cases, fixation. The anterior facet of C1 becomes locked on the facet of C2, causing impaired rotation at this joint. It can occur with or without C1-C2 dislocation [6,7].

The entity of atlanto-axial dislocation was first described by Corner [8] who reviewed 20 cases. Since then there have been a remarkable number of cases of this not uncommon and potentially catastrophic condition [9-11]. Chiapparini et al. [12] described atlanto-axial rotatory fixation in four pediatric cases, as a rare cause of torticollis that may occur spontaneously or in association with trauma or upper respiratory tract infection. Subluxation has also been described following retropharangeal abscess, tonsillectomy, or pharyngoplasty [13,14]. Other forms of atlanto-axial dislocation develop following acute cervical trauma or due to slow erosion around the joints in, for example rheumatoid arthritis, ankylosing spondylitis, and tubercular arthritis [13].

Fielding and Hawkins [15,16], studied a series of seventeen cases. All patients had torticollis and a diminished range of movement. The typical head position was lateral flexion to one side, rotation toward the opposite side and slight flexion – the " cock robin" position. None of the reported cases manifested other clinical and or radiological features in favor of a syndromic association.

Hertzka et al., [1], described atlanto-axial rotatory dislocation in a series of three patients with Marfan syndrome. Two of his patients developed acute torticollis postoperatively, following pectus excavatum repair. The diagnosis was made in the third patient after she presented to the emergency room with a weeklong history of unresolved neck pain, following minor trauma. Hobbs et al., [17] described the diagnostic criteria in Marfan syndrome.

The vertebral defects encountered in SSS consist mainly of fusion of multiple vertebral bodies in the cervical, thoracic, and lumbar spine (block vertebrae) together with the fusion of the posterior elements of the neural arch. Thoracic scoliosis is the most common presentation of this disorder [18-23]. There are some reports with additional cervical abnormalities; Seaver et al., [3] reported a female child presented with subluxation of C2-C3. Two other patients reported by Langer et al. [21] manifested odontoid hypoplasia only. Neither AARF nor visualization of the craniocervical junction by CT scan has been reported in association with SSS.

The cervical spine differs from the thoracic or lumbar region in both anatomy and functional respects. In particular the upper cervical spine has its own unique anatomy, including several ligaments designed to permit axial rotation of the atlas and head. Therefore the pathogenesis of cervicothoracic scoliosis should be carefully evaluated.

Based on the present study, we suggest that the mechanism of the rotatory dislocation of C1-C2 is due to the existence of two adverse factors. First, the presence of a unilateral cervical unsegmented bar. Second, the congenital ligamental laxity which possibly caused further injury to the poor ligamental fixation of the scoliotic cervical region, and specifically the atlas-axis complex. We believe that the craniocervical junction is a vulnerable and sensitive area needs detailed evaluation in patients with congenital scoliosis.

## Conclusion

1) The classical applied methodology of studying scoliotic patients should be modified in accordance with unusual findings. Particular attention and prompt assessment should be paid to other associated anomalies such as; unusual phenotypic features, musculoskeletal ligamentous hyperlaxity/articular stiffness, small hands or fingers (brachydactyly)/unusual long fingers (arachnodactyly), unusual long arm span (dolichostenomelia)/unusually short arms and or forearms (rhizomelia).

2) We wish to stress on the significant role of CT scan as a diagnostic tool in the interpretation of different bone malformation complexes.

To our knowledge there have been no reports describing such changes in association with (SSS).

## Abbreviations

(SSS) Spondylocarpotarsal synostosis syndrome; (AARF) Atlanto-axial rotatory fixation; (OFC) Occipital-Frontal-Circumference; (Gravida 3 abortus 0) mother had 3 children and no history of spontaneous abortions.

## Competing interests

The author(s) declare that they have no competing interests.

## Authors' contributions

Ali Al Kaissi: Authors own work. Was responsible for a) writing the MS, b) conception and design, and c) analysis of data.

Farid Ben Chehida, Hassan Gharbi and Maher Ben Ghachem: Participated in analysis of data.

Franz Grill and Klaus Klaushofer: FG.

**Figure 2:**
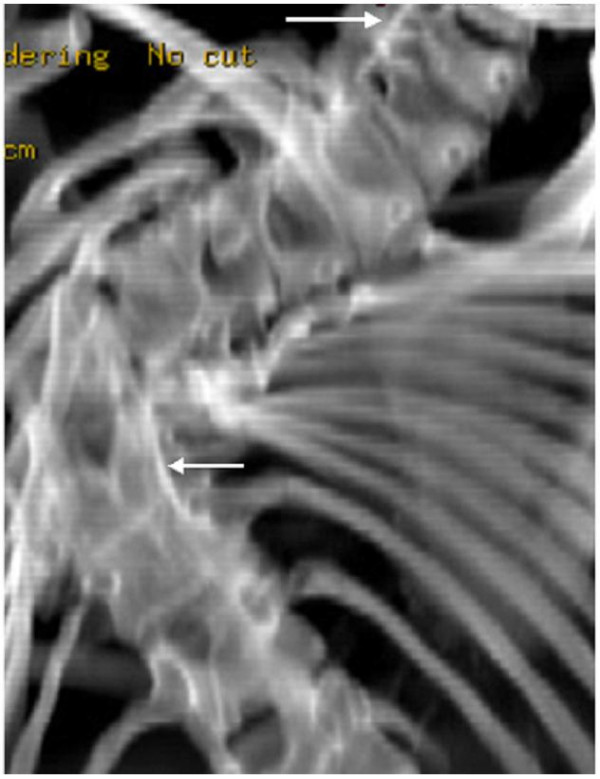
showed spinal segmentation defects and marked thoracic scoliosis (Cobb angle of 85 degree) with a unilateral unsegmented bar along the cervical and the thoracic spine, CT scan showed two unsegmented spinal bars-upper right arrow showed unsegmented unilateral cervical bar at the level of the C5-C6. Lower left arrow showed another unilateral unsegmented bar extending from T6-T10.

**Figure 3:**
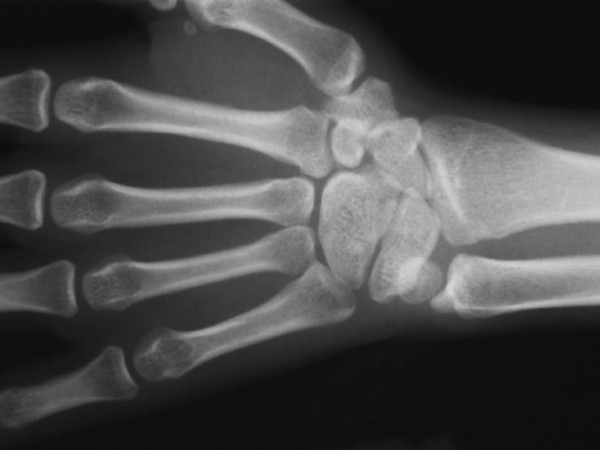
Hand x-ray showed synostosis between capitate-hamate-and lunate-triquetrum.

**Figure 4:**
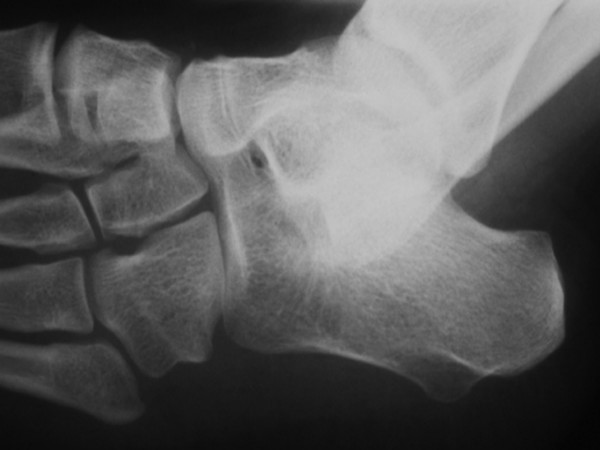
Foot x ray showed multiple fusions, talo-calcanear, talo-navicular, naviculo-calcanear, cunieform2-cuneiform 3.

**Figure 5:**
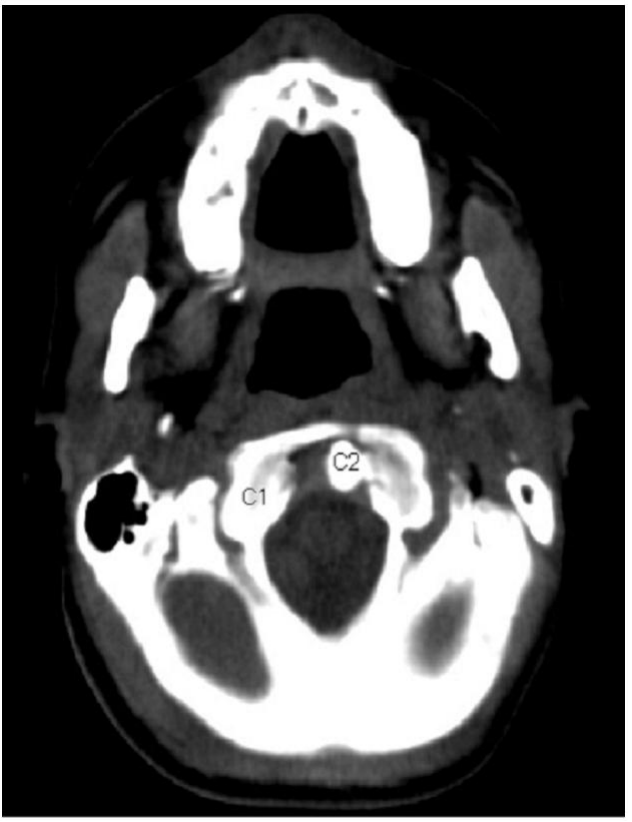
Axial CT image demonstrated AARF; the right lateral mass of the atlas C1 lies anterior to the articular surface of C2.

**Figure 6:**
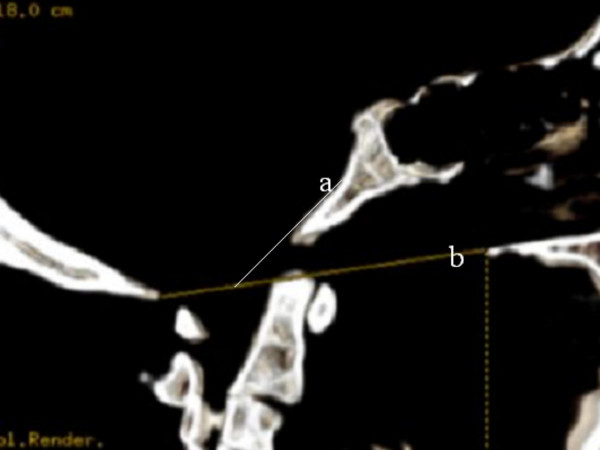
Sagittal CT image reveals anterior displacement of the odontoid process (O) to the posterior aspect of the anterior arch of C1. Note the Wachenheim clivus line* (a), which is drawn along the posterior aspect of the clivus toward the odontoid process-in our patient – the line does not intersect and is not tangential to the odontoid process-this confirms the abnormality and indicates the existence of occipito-atlas subluxation. The Chamberlain's line **(b) showed no associated basilar invagination.

**Figure 7:**
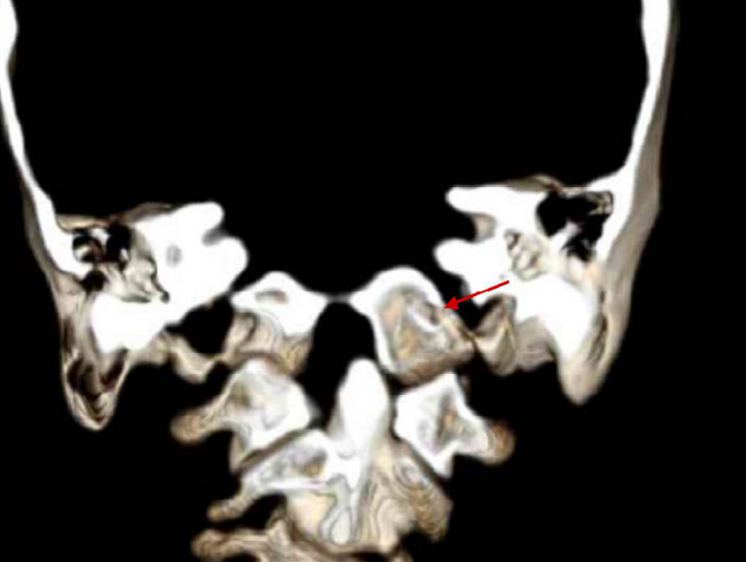
Coronal computed tomographic reconstructions demonstrating dislocation at C1-C2 and Subluxation of the occipito-atlas junction. Note C2 in the coronal position with the anterior arch of C1 overlying the left facet. Sphenoide bone, anterior part of occipital foramen (arrow).
